# Biofilm Formation by Rice Rhizosphere Nitrogen-Fixing Microorganisms and Its Effect on Rice Growth Promotion

**DOI:** 10.3390/biology14091249

**Published:** 2025-09-11

**Authors:** Jae-Hyeon Oh, Eunhee Kim, Mihyun Cho

**Affiliations:** Supercomputing Center, Department of Agricultural Biotechnology, National Institute of Agricultural Sciences, Rural Development Administration, 370, Jeonju-si 54874, Jeollabuk-do, Republic of Korea; kehrda@korea.kr (E.K.); chomi1@korea.kr (M.C.)

**Keywords:** biofilm formation, nitrogen-fixing microorganisms, plant growth promotion, rice, sustainable agriculture

## Abstract

Excessive nitrogen fertilizer use contributes to environmental pollution and undermines agricultural sustainability. Enhancing symbiotic interactions between rice and nitrogen-fixing microorganisms offers a promising strategy to potentially improve nitrogen use efficiency (NUE). This study investigates the role of rice root exudates in promoting biofilm formation by nitrogen-fixing microbes to enhance nitrogen fixation. Nine nitrogen-fixing microbial strains were evaluated for biofilm formation in response to flavone and apigenin treatments, with *Gluconacetobacter diazotrophicus* KACC 12358 serving as the reference strain. The most responsive strain was selected, and a library of 1597 natural compounds was screened to identify those that promote biofilm formation in both the selected and reference strains. *A. indigens* KACC 11682 exhibited the highest biofilm-forming capacity, with apigenin treatment showing an OD595 value approximately 1.4 times higher than the DMSO control. Screening identified 68 compounds that enhanced biofilm formation by more than 500% compared to the control. Among them, eight compounds induced strong biofilm formation (O.D. > 2.0) in *A. indigens*. Cardamomin, a chalconoid flavonoid, emerged as one of the most effective compounds, showing a 245% increase in biofilm formation. Growth promotion assays showed that *A. indigens* increased rice fresh weight by approximately 128% compared to untreated controls. This study demonstrates the potential of rice root exudate-derived compounds to promote beneficial symbiosis with nitrogen-fixing microbes. These findings offer a novel approach that may contribute to enhancing rice NUE. Future research will focus on evaluating the long-term effects of these compounds and microorganisms, assessing their applicability in real agricultural settings, and conducting further validation across various rice cultivars.

## Simple Summary

Improving nitrogen use efficiency (NUE) in rice cultivation is crucial for sustainable agriculture and for reducing environmental pollution caused by excessive fertilizer use. In this study, we found that certain beneficial microorganisms living in the rice rhizosphere can form strong biofilms when exposed to specific natural compounds—particularly cardamomin, a flavonoid found in various plants. Among the tested strains, *Azoarcus indigens* KACC 11682 exhibited the highest biofilm-forming ability and significantly promoted rice growth, increasing biomass by approximately 128%. Through the screening of 1597 natural compounds, 68 were identified as strong inducers of biofilm formation. These findings suggest that enhancing the symbiosis between rice and nitrogen-fixing microbes using targeted compounds can reduce the need for synthetic fertilizers and support more eco-friendly rice farming.

## 1. Introduction

Modern agriculture faces the critical challenge of maintaining high productivity to meet the food demands of a rapidly growing global population. Nitrogen fertilizers play an essential role in this process; however, their excessive use has led to serious environmental problems [1]. When nitrogen is applied beyond the absorption capacity of crops, it is converted into potent greenhouse gases such as nitrous oxide (N_2_O) and released into the atmosphere [2]. Nitrous oxide possesses a global warming potential approximately 298 times greater than that of carbon dioxide (CO_2_) and thus significantly contributes to climate change [3]. Additionally, nitrate (NO_3_^−^) leaching contaminates water bodies, threatening aquatic ecosystems and compromising groundwater quality, which can be detrimental to drinking water supplies [4]. The global agricultural sector is increasingly focusing on harnessing plant-microbe interactions to enhance crop productivity and reduce dependence on synthetic fertilizers [5].

To address these issues, it is globally imperative to maintain agricultural productivity while reducing nitrogen fertilizer inputs. Improving nitrogen use efficiency (NUE) has emerged as a key strategy to achieve this goal [6]. Enhancing the NUE of staple crops such as rice is particularly critical for ensuring global food security and protecting the environment [7]. Previous studies, including those on genetic engineering, conventional breeding, and the application of plant growth-promoting rhizobacteria (PGPR), have explored various approaches to improve rice NUE. Among these, leveraging symbiotic interactions between rice and nitrogen-fixing microorganisms has gained attention as an environmentally sustainable and effective solution [8].

Root exudates secreted by rice plants play a pivotal role in promoting such symbiotic interactions [9]. These exudates stimulate the growth and biofilm formation of nitrogen-fixing microorganisms, thereby facilitating biological nitrogen fixation and enhancing nitrogen availability to the plant [10,11]. Recent meta-analyses have highlighted how microbial biofilms can improve nutrient uptake efficiency and root health, significantly impacting plant growth outcomes [12]. While the importance of root exudates in stimulating microbial growth and biofilm formation is known, the specific identification of novel, potent biofilm-inducing compounds from a large natural library, particularly those relevant to rice, remains an underexplored area [13,14]. The application of naturally derived compounds to manipulate rhizosphere microbiomes is being actively explored as a sustainable agricultural practice [15]. Moreover, advances in genomics are enabling the breeding of rice varieties with improved nitrogen use traits, often in conjunction with beneficial root-associated microbes [16]. Understanding the regulatory mechanisms of biofilm formation presents new opportunities for designing tailored microbial consortia for field application [17]. Therefore, identifying exudate-derived compounds that favor nitrogen-fixing microorganisms is critical for improving NUE in rice. This study addresses this gap by systematically identifying novel biofilm-inducing compounds that can enhance beneficial plant-microbe interactions for improved rice NUE. Our originality lies in the comprehensive screening and selection of *A. indigens* KACC 11682 as a novel and highly effective biofilm-forming nitrogen-fixing microorganism, and the large-scale screening of 1597 natural compounds to identify strong biofilm inducers, including the chalconoid cardamomin. This integrated approach, linking microbial biofilm formation with plant growth promotion, offers a novel and sustainable alternative to conventional fertilizer reduction strategies. Furthermore, we plan to investigate the biosynthetic pathways of the selected compounds and employ gene editing technologies to enhance their secretion in rice roots, thereby maximizing beneficial plant-microbe interactions. The compounds identified in this study have the potential to contribute to rice breeding efforts focused on improving NUE. Ultimately, this approach is expected to reduce nitrogen fertilizer usage and support the development of sustainable agricultural systems. Compared to conventional fertilizer reduction strategies, our method offers a more sustainable and long-term solution. Moreover, the findings of this study could be extended to other major cereal crops, contributing to a paradigm shift in global agricultural practices.

## 2. Materials and Methods

### 2.1. Selection of Nitrogen-Fixing Microorganisms

To identify microbial strains capable of enhancing nitrogen fixation efficiency through symbiosis with rice, nine nitrogen-fixing microorganisms were obtained from the Korean Agricultural Culture Collection (KACC), https://genebank.rda.go.kr (accessed on 4 June 2025) (Table 1). Among them, *G. diazotrophicus* KACC 12358 was selected as a reference strain, based on the findings of [18], which reported its superior nitrogen-fixing ability through biofilm formation. The microorganisms were selected from genera known for nitrogen fixation, choosing species available in the KACC repository.

### 2.2. Identification of Strains Using 16S rRNA Sequence Information

For microbial identification, 16S rRNA sequence information was utilized. Sequence data were retrieved from the NCBI database, https://www.ncbi.nlm.nih.gov (accessed on 4 June 2025). Sequence alignment was performed using MEGA 11 [19], and unique, non-redundant regions were identified. Based on these regions, primers were designed using the Primer3 program(ver. 4.1.0), https://primer3.ut.ee (accessed on 4 June 2025).

### 2.3. Analysis of Microbial Growth Characteristics

To evaluate the growth characteristics of each nitrogen-fixing microorganism, the optimal growth medium was first selected among NA, R2A, and RAE media. To determine colony-forming units (CFU), samples were serially diluted, plated onto solid media, and incubated under appropriate conditions. After incubation, the number of colonies was counted, and CFUs were calculated considering the dilution factors. Specifically, precultures were adjusted to an OD600 of 0.3 using a spectrophotometer, followed by serial dilutions up to 10^−9^. Dilutions from 10^−5^ to 10^−9^ were plated onto solid media, with each dilution divided into five sections per plate and 20 µL aliquots dispensed into each section. Each condition was prepared in triplicate. After plating, the plates were air-dried under sterile conditions before incubation at optimal temperatures, and colony formation was monitored.

Growth curves of each microorganism were also determined by measuring OD600 at the early, mid, and late stages of cultivation. Specifically, precultures were adjusted to an OD600 of 0.01, and 200 µL of each culture was inoculated into a 96-well plate, with three replicates per strain. Cultures were incubated for up to 96 h, and growth was monitored over time to assess proliferation rates and growth patterns under the tested conditions [20].

### 2.4. Acquisition of Natural Compound Library

To identify compounds that enhance biofilm formation by nitrogen-fixing microorganisms from rice root exudates, a total of 1597 natural compounds were obtained from the Korea Chemical Bank, https://chembank.org (accessed on 4 June 2025) (Supplementary Materials Table S1). These compounds were prepared at a concentration of 5 mM and were used for screening to evaluate their effects on biofilm formation.

### 2.5. Evaluation of Biofilm Formation Ability of Nitrogen-Fixing Microorganisms Using Flavone Compounds

To evaluate the biofilm formation ability of nitrogen-fixing microorganisms, the known biofilm-inducing compounds flavone, apigenin, and luteolin were each applied at 2 µL per well, and biofilm formation was assessed using the crystal violet staining method. Specifically, each microorganism (OD600 = 0.01) was inoculated into a 96-well plate with 198 µL of culture medium, followed by the addition of 2 µL of each compound to designated wells. After three days of incubation, the culture medium was removed, and the plate was fixed and stained with 0.2% crystal violet solution. After staining, the residual dye was removed, the wells were washed with distilled water, and the biofilms were dissolved in 95% ethanol. The absorbance was measured at OD595 to quantify the degree of biofilm formation [18]. Through this process, nitrogen-fixing microorganisms with superior biofilm formation ability were selected.

### 2.6. Screening of Biofilm Formation in Nitrogen-Fixing Microorganisms upon Natural Compound Treatment

Each compound from the natural compound library was prepared at a concentration of 5 mM and added at 2 µL per well to the microbial cultures to evaluate its effect on biofilm formation. The microorganisms used for screening included the selected strains as well as the control strain, *G. diazotrophicus* KACC 12358. Screening was performed in 96-well plates, and cultures were incubated for 48 h. Biofilm formation was quantified using the crystal violet staining method in 96-well polystyrene microtiter plates (Corning Inc., Corning, NY, USA), and absorbance at 595 nm was measured using a microplate reader (Multiskan™ FC, Thermo Fisher Scientific, Waltham, MA, USA). Based on these measurements, the top 50 compounds promoting the highest levels of biofilm formation in the selected strains were initially selected.

### 2.7. Final Selection of Compounds

Among the 50 compounds selected from the primary screening, compounds were finally selected based on their ability to significantly induce biofilm formation in *A. indigens* KACC 11682. To accurately identify top-performing compounds, all raw optical density values were normalized against the mean of the DMSO controls for *A. indigens* on the same plate. The relevance of these compounds to known rice metabolic pathways was also considered. These compounds are expected to enhance nitrogen use efficiency (NUE) in rice and will be utilized in future studies to strengthen the symbiotic interaction between rice and nitrogen-fixing microorganisms.

### 2.8. Preliminary Test of Nitrogen-Fixing Microorganisms on Rice Growth Promotion

To evaluate the effect of the selected microorganisms on rice growth, each microorganism was inoculated into Yoshida nutrient solution [21] with controlled nitrogen sources. The experiment was conducted on the Samgwang rice variety, and after 70 days of inoculation, plant height, biomass, and the EC and pH of the Yoshida solution were measured. The experiment was repeated 10 times, and the results were averaged.

### 2.9. Statistical Analysis

All experiments were conducted with at least three independent biological replicates, and data are presented as the mean ± standard deviation (SD). Statistical analyses were performed using R statistical software (version 4.2.2, R Core Team, Vienna, Austria) or SAS (version 9.4, SAS Institute Inc., Cary, NC, USA). Differences between multiple groups were analyzed using one-way analysis of variance (ANOVA) followed by Tukey’s honestly significant difference (HSD) post hoc test for multiple comparisons. For comparisons between two groups, Student’s *t*-test was used. A *p*-value < 0.05 was considered statistically significant.

## 3. Results

### 3.1. Optimization of Cultivation Conditions and Microbial Identification Results

In this study, the optimal cultivation conditions for 9 nitrogen-fixing microorganisms were selected by comparing cultivation on NA, R2A, and RAE media. The growth characteristics of the strains on each medium were evaluated, and the results showed that most strains exhibited the most vigorous growth on R2A medium, particularly *A. indigens* KACC 11682 and Herbaspirillum strains, which displayed the most prominent growth effects. Based on these results, R2A medium was selected as the most suitable medium for the cultivation of the 9 nitrogen-fixing microorganisms. After selecting the optimal medium, each strain was cultured on R2A medium, and CFU (OD600 = 0.3) was measured to quantitatively assess the growth. The CFU calculation showed that the growth of all strains was higher on R2A medium compared to other media, suggesting that this medium provides the most suitable environment for microbial proliferation and colony formation (Supplementary Materials Figure S1).

Microbial identification was performed using 16S rRNA sequencing, and bands of the expected size were confirmed for all strains (Supplementary Materials Figure S2). This indicates that successful 16S rRNA-based identification of each strain was achieved, supporting the purity and accuracy of the microbial identification. These results demonstrate the successful establishment of optimal cultivation conditions for the 9 nitrogen-fixing microorganisms used in the study and the successful identification of the microorganisms.

### 3.2. Growth Curve Analysis

The growth curves of the 9 nitrogen-fixing microorganisms were analyzed, and the results showed that *A. indigens* KACC 11682 exhibited stable growth at 37 °C, while the other 8 strains grew stably at 30 °C (Supplementary Materials Figure S3). In particular, *H. frisingense* KACC 15012 showed the fastest growth rate, reaching its peak OD600 value after 24 h. In contrast, *A. indigens* KACC 11682 showed slower growth. This was in contrast to the visual observation, where a large amount of suspended material, such as biofilm, was observed. The *G. diazotrophicus* KACC 12358 strain, which had previously demonstrated nitrogen fixation and biofilm formation ability in the study by [18], showed a growth pattern similar to that of *H. frisingense* KACC 15012, but with relatively slower growth.

### 3.3. Evaluation of Biofilm Formation Ability for Selection of Nitrogen-Fixing Microorganisms with High Biofilm Formation Ability

**Figure 1:**
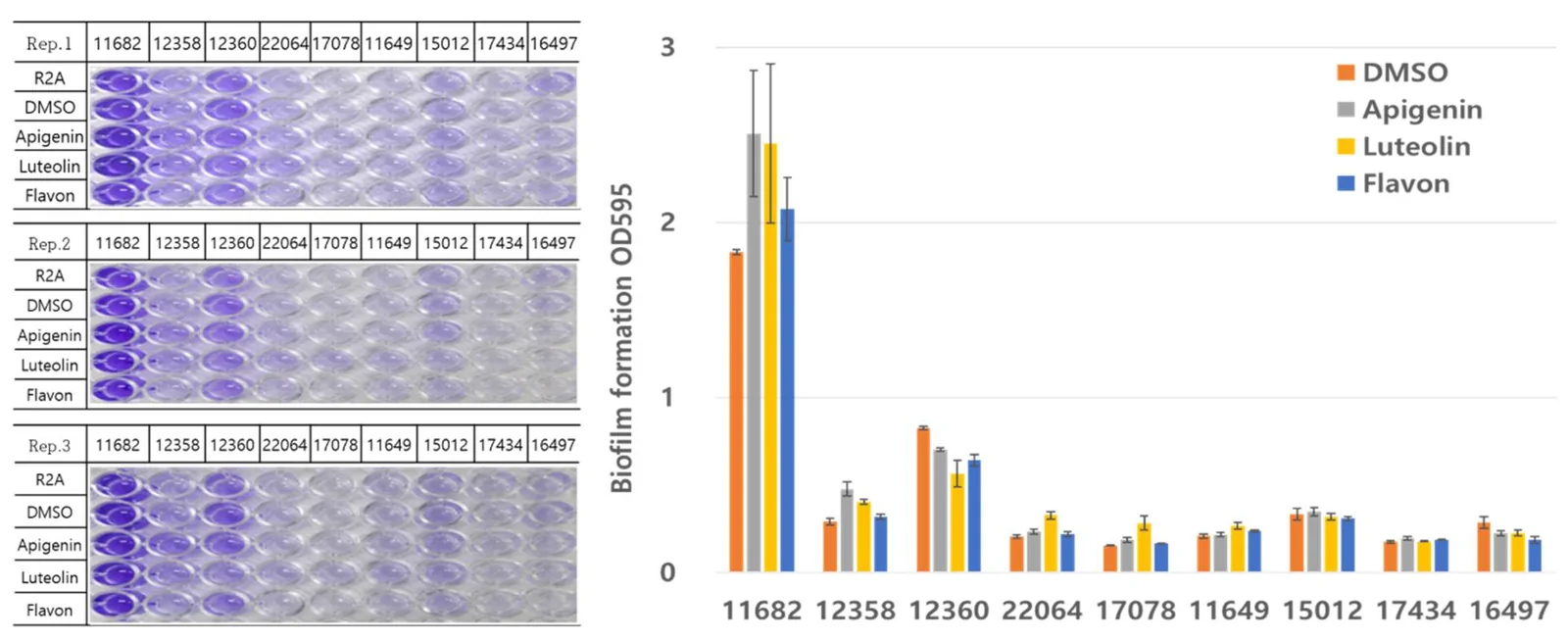
Biofilm formation of nine nitrogen-fixing microorganisms upon treatment with flavone compounds. Biofilm formation was quantified by crystal violet staining (OD595) after three days of incubation. Treatments include control (DMSO), apigenin, luteolin, and flavone. Data represent the mean ± standard deviation of three independent replicates. Statistical differences were assessed using one-way ANOVA followed by Tukey’s HSD (*p* < 0.05).

The biofilm formation ability of 9 nitrogen-fixing microorganisms was evaluated using the crystal violet staining method. The results showed that *A. indigens* KACC 11682 exhibited the highest biofilm formation ability in all treatments, including the control (DMSO), apigenin, luteolin, and flavone (Figure 1). *A. indigens* KACC 11682 showed an OD595 value approximately 1.4 times higher with apigenin treatment compared to the control (DMSO). When compared to the control strain, *G. diazotrophicus* KACC 12358, the apigenin-treated *A. indigens* KACC 11682 demonstrated more than a 5-fold higher biofilm formation ability. In relative comparison, the biofilm formation abilities were ranked as follows: *A. indigens* KACC 11682, *G. liquefaciens* KACC 12360, and *G. diazotrophicus* KACC 12358. All compounds were dissolved in dimethyl sulfoxide (DMSO; Sigma-Aldrich, St. Louis, MO, USA) prior to treatment.

### 3.4. Natural Compound Screening Results

**Figure 2:**
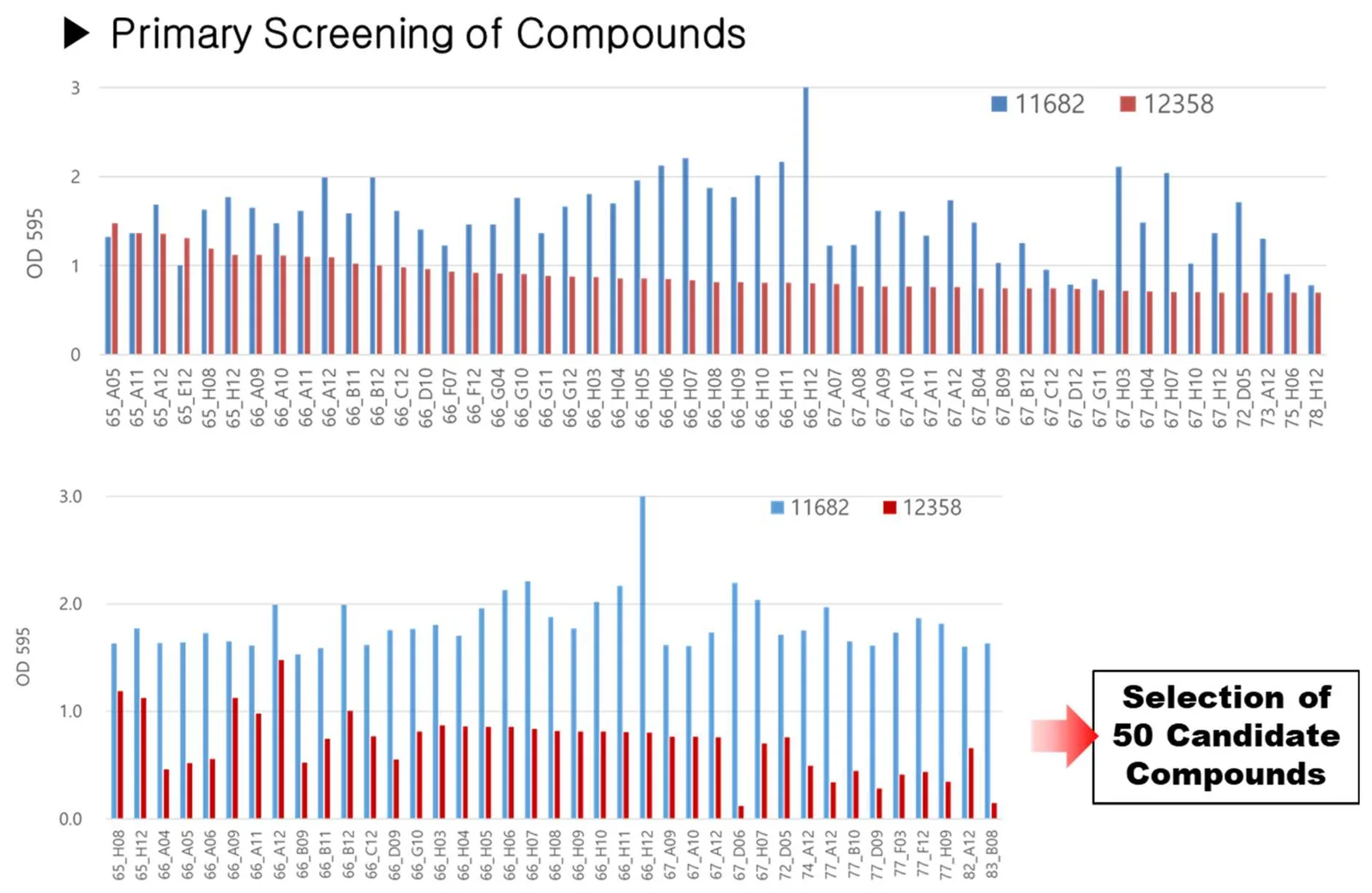
Screening process and partial results of biofilm formation on 1597 natural compounds. The figure illustrates the high-throughput screening process and shows the top 50 compounds that significantly enhanced biofilm formation in *A. indigens* KACC 11682. Biofilm formation was quantified by crystal violet staining (OD595) after 48 h of incubation. The percentage increases are relative to the *A. indigens* DMSO-treated control. Data represent the mean ± standard deviation of three independent replicates. Among the compounds evaluated, which included alkaloids and flavonoids known to be present in various plants, cardamomin was a representative compound showing high activity. This compound showed a 245% increase in relative comparison. Cardamomin was selected from the flavonoid group due to its position at the top of the biosynthetic pathway, which strategically aligns with ongoing research on chalcones in flavonoid biosynthesis. Furthermore, it was ranked fifth in biofilm formation ability when tested alone in *A. indigens* KACC 11682, indicating a relatively high level of activity.

As shown in Figure 2, screening of 1597 natural compounds revealed the top 50 compounds that most significantly enhanced biofilm formation in *A. indigens* KACC 11682. The reported percentage increases for these compounds (e.g., 245%, 500%, 1793%) were calculated relative to the *A. indigens* KACC 11682 control group treated solely with DMSO, indicating their specific stimulative effect on *A. indigens*’s biofilm formation (Supplementary Materials Table S2). While many selected compounds also showed higher absolute biofilm formation than the reference strain, *G. diazotrophicus* KACC 12358, our primary selection criterion was the induction effect within *A. indigens* KACC 11682. For example, the compound with the highest activity, oxytetracycline hydrochloride, increased biofilm formation by 1793% compared to the DMSO control. This compound, known as an antibiotic synthesized by actinomycetes, was identified in this study as a biofilm-promoting substance.

Ultimately, the compounds identified in this study are considered to have high potential for improving nitrogen use efficiency (NUE) in rice. Additionally, these compounds demonstrated a strong association with specific rice gene pathways, and they are expected to play a key role in enhancing the symbiotic interaction between rice and nitrogen-fixing microorganisms in future research.

### 3.5. Effect of Nitrogen-Fixing Microorganisms on Rice Growth

**Figure 3:**
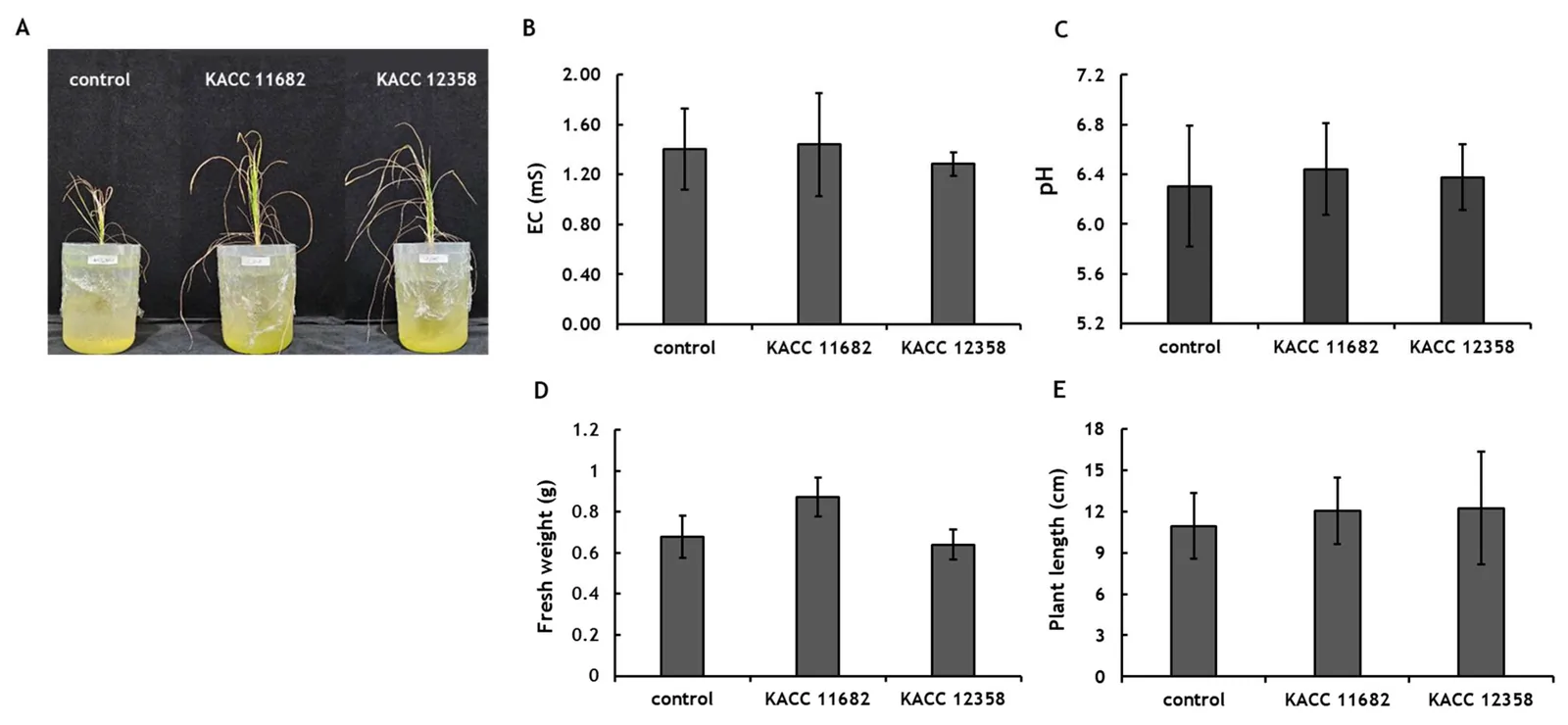
Effect of nitrogen-fixing bacterial inoculation on rice seedlings grown in nitrogen-free hydroponic solution. Seedlings were cultivated in N-free nutrient solution to evaluate growth responses attributable to biological nitrogen fixation by the inoculated strains. (**A**) Representative images at 14 days post-inoculation (left to right): uninoculated control; inoculated with Azospirillum indigens KACC 11682; inoculated with Gluconacetobacter diazotrophicus KACC 12358. (**B**) Electrical conductivity of the nutrient solution (EC, mS/cm). (**C**) pH. (**D**) Fresh weight (g). (**E**) Plant length (cm). Bars indicate mean ± SD (sample sizes as described in Methods). Group differences were tested by one-way ANOVA followed by Tukey’s HSD (*p* < 0.05).

In the growth promotion test using the Samgwang rice variety, rice inoculated with *A. indigens* KACC 11682 showed approximately a 128% increase in biomass compared to the untreated control group (Figure 3). Furthermore, rice inoculated with *A. indigens* KACC 11682 exhibited a trend of about 136% greater biomass than rice inoculated with *G. diazotrophicus* KACC 12358. However, a contrasting trend was observed in plant height, with rice inoculated with *G. diazotrophicus* KACC 12358 showing about a 112% increase in plant height compared to the control, though the difference was not statistically significant.

## 4. Discussion

In this study, we evaluated the plant growth-promoting potential of *A. indigens* KACC 11682, a known nitrogen-fixing microorganism. The strain markedly promoted plant growth, increasing fresh weight by 128%. However, because a nitrogen-rich Yoshida solution was used in our experiments, nitrogenase activity would be expected to be largely suppressed, indicating that the observed effects cannot be attributed directly to enhanced nitrogen use efficiency (NUE) through biological nitrogen fixation alone.

Instead, the significant growth promotion is likely attributable to a combination of PGPR mechanisms beyond nitrogen fixation. These include phytohormone production (auxins, gibberellins, cytokinins), which influence root development and nutrient uptake; nutrient solubilization (e.g., phosphate and potassium); and indirect effects such as pathogen suppression via antibiosis or induced systemic resistance. Notably, *A. indigens* exhibited a significantly greater capacity for biofilm formation than previously studied strains such as *G. diazotrophicus* KACC 12358. This robust biofilm formation is crucial for effective root colonization and for establishing strong plant-microbe interactions that facilitate multiple PGPR mechanisms.

Our large-scale screening identified 68 natural compounds that significantly enhanced biofilm formation in *A. indigens*. Among these, cardamomin, a flavonoid, showed the most promising results, yielding a 245% increase in biofilm formation. While direct evidence for cardamomin de novo biosynthesis across all rice cultivars requires further investigation, flavonoids are well-established plant secondary metabolites involved in plant-microbe signaling. The strong biofilm-inducing activity of these compounds suggests an important role in enhancing symbiotic interactions between rice and nitrogen-fixing microorganisms.

The significant increase in rice biomass following *A. indigens* inoculation strongly suggests the strain’s contribution to rice growth, which may lead to potential improvements in NUE. These findings align with recent research highlighting the multifaceted roles of PGPR in sustainable agriculture and underscore the potential of leveraging targeted natural compounds to strengthen symbiotic interactions with nitrogen-fixing microorganisms, thereby improving rice NUE and reducing reliance on synthetic fertilizers.

Future studies will rigorously investigate the synergistic effects of *A. indigens* and top biofilm-inducing compounds (particularly cardamomin) on rice growth and NUE under various conditions, including field trials. This will involve practical application methods such as seed treatment or root drenching. Furthermore, direct quantification of biological nitrogen fixation via 15N2 incorporation assays under nitrogen-limiting conditions will be crucial to fully elucidate the contribution of nitrogen fixation. Additionally, verification of the chemical screening results by reordering and retesting top hits from different sources represents a critical next step to confirm reproducibility and reliability.

## 5. Conclusions

This study successfully identified *A. indigens* KACC 11682 as a novel and highly effective strain capable of forming robust biofilms and significantly promoting rice growth, thereby enhancing symbiotic interactions and showing potential for improving nitrogen use efficiency (NUE).

Furthermore, the systematic screening of natural compounds led to the discovery of potent biofilm inducers, such as cardamomin, demonstrating their potential to strengthen these beneficial plant-microbe interactions. These findings offer a promising and sustainable strategy to improve rice NUE and reduce environmental impacts associated with excessive nitrogen fertilizer use.

Future research will focus on rigorously evaluating the synergistic effects of *A. indigens* and the identified compounds on rice growth and NUE in controlled and field environments, including direct quantification of biological nitrogen fixation. Further validation of the screening results and assessment of applicability across diverse rice cultivars will be crucial for developing concrete strategies for sustainable agricultural practices.

## Figures and Tables

**Table 1:** Information on nitrogen-fixing microorganisms obtained from the Agricultural Microorganism Bank (KACC).

Source	Diazotroph Inoculant	KACC
Rice	*Azoarcus indigens*	11682
	*Gluconacetobacter diazotrophicus*	12358
	*Gluconacetobacter liquefaciens*	12360
	*Gluconacetobacter liquefaciens*	22064
	*Gluconacetobacter* sp.	17078
	*Herbaspirillum chlorophenolicum*	11649
	*Herbaspirillum frisingense*	15012
	*Herbaspirillum rhizosphaerae*	17434
	*Herbaspirillum* sp.	16497

## Data Availability

The original contributions presented in this study are included in the article/supplementary material. Further inquiries can be directed to the corresponding author.

## Supplementary Materials

The following supporting information can be downloaded at: https://www.mdpi.com/article/10.3390/biology14091249/s1, Figure S1: Optimal cultivation conditions and CFU determination for nitrogen-fixing microorganisms, Figure S2: Design and validation of specific primers for species identification within the 16S rRNA region, Figure S3: Growth curve determination for each nitrogen-fixing microorganism, Table S1: Information on 1597 natural compounds obtained from the Korean Compound Bank, Table S2: Biofilm formation levels of 1597 natural compounds.
