# Supplementary material for: Biofilm Formation by Rice Rhizosphere Nitrogen-Fixing Microorganisms and Its Effect on Rice Growth Promotion
Source: Biology (Basel). 2025 Sep 11;14(9):1249. doi: 10.3390/biology14091249 (PMC12467018; doi:10.3390/biology14091249)
Supplement: Supplementary file 1 [file biology-14-01249-s001.zip › Biology_JaeHyeon Oh_Supplemenary Figures and Tables_Styled.pdf]

Supplementary Information

Title:

Biofilm Formation by Rice Rhizosphere Nitrogen-Fixing Microorganisms and Its Effect on Rice Growth Promotion

Authors:

Jae-Hyeon Oh[1,*], Eunhee Kim[1], Mihyun Cho[1]

Affiliation:

[1]National institute of Agricultural Sciences, Rural Development Administration, 370, Jeonju-si, Jeollabuk-do 54874, Korea.

*Corresponding author: Jae-Hyeon Oh

E-mail: jhoh8288@korea.kr

Supplementary Figures

KACC	NA	R2A		RAE
	36hr	36hr	56hr	36hr
11682	+	+ +	+ + +	-
12358	-	+	+ +	-
12360	-	+	+ +	-
22064	+	+ +	+ + +	+ +
17078	+ +	+ + +	+ + +	+ +
11649	+	+ +	+ + +	-
15012	+ +	+ + +	+ + +	+
17434	+ +	+ + +	+ + +	-
16497	+	+ + +	+ + +	-

KACC	CFU
11682	3×10^{6}
12358	1×10^{9}
12360	5×10^{6}
22064	6.3×10^{10}
17078	8×10^{5}
11649	1×10^{7}
15012	2×10^{6}
17434	1×10^{7}
16497	1×10^{6}

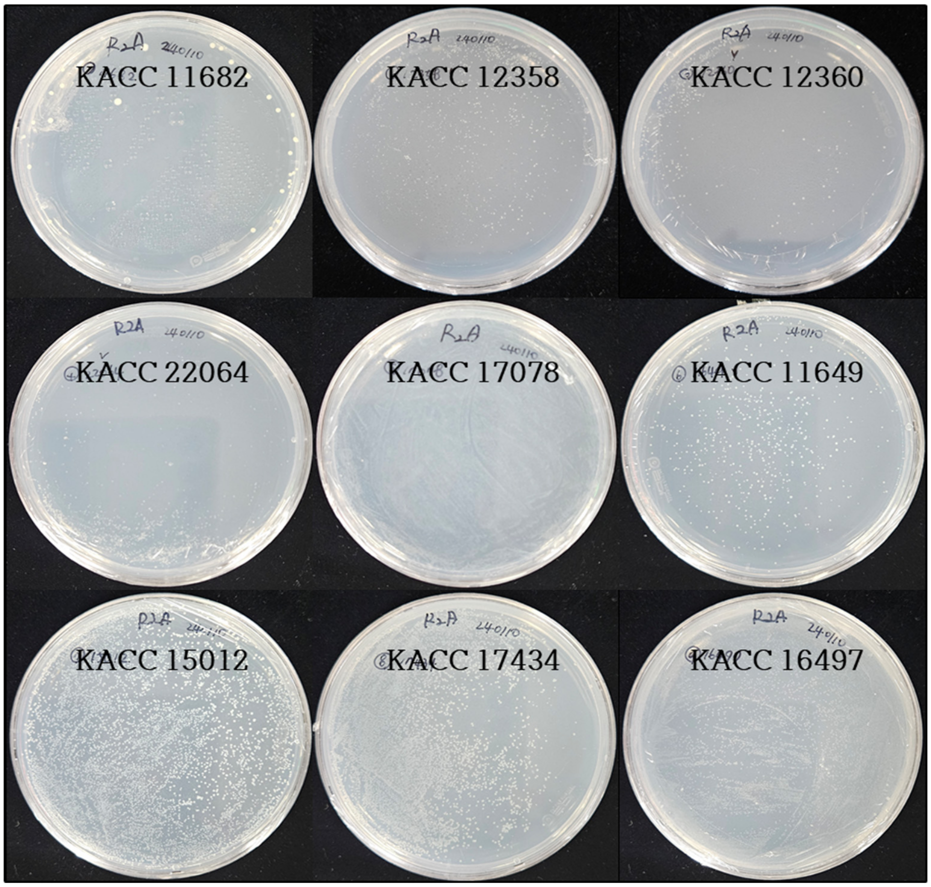

Figure S1 Optimal cultivation conditions and CFU determination for nitrogen-fixing microorganisms.

No.	KACC		Primer	Size(bp)
1	11682	F	TCGGCCAATACCCGAAG	561
		R	GCTCTCACCAAGGTT	
2	12358	F	ACAGACTAGAGTGTGAG	373
		R	ATCTCTGACTGCAGC	
3	12360	F	CCAAACTAGAGTGTGAG	373
		R	ATCTCTGGATACAGC	
4	22064	F	CCAAACTAGAGTGTGAG	373
		R	ATCTCTGGATACAGC	
5	17078	F	ATAGTCTAGAGTGAGGA	200
		R	CAGCGTCAGTAAGGA	
6	11649	F	GCAGCTAATATCTGCGA	559
		R	AATCTCTTCAGGATT	
7	15012	F	CTGGATAATACCTGGAG	559
		R	AATCTCTTCGGGATT	
8	17434	F	GAAGCTAATATCTTTGC	559
		R	AATCTCTTCGGCCTT	
9	16497	F	CGATCTAATACATTGGG	163
		R	GATCTCTCCAAGCTT	

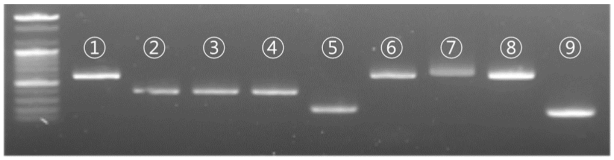

Figure S2 Design and validation of specific primers for species identification within the 16S rRNA region.

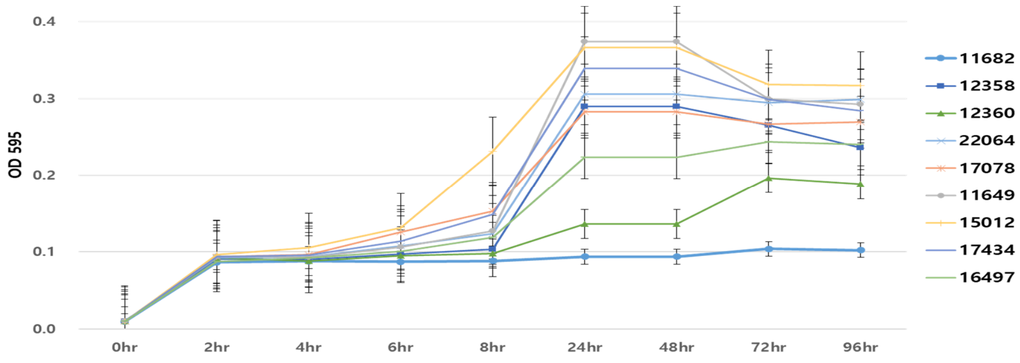

Figure S3 Growth curve determination for each nitrogen-fixing microorganism.

Supplementary Tables

Table S1 Information on 1,597 natural compounds obtained from the Korean Compound Bank.

Table S2 Biofilm formation levels of 1,597 natural compounds.
